# Empowering Caregivers of People with Dementia in India: Addressing Financial, Emotional, and Policy Gaps

**DOI:** 10.1002/alz70858_106761

**Published:** 2025-12-24

**Authors:** Niharika Dadoo, Ajay Prakash, Bikash Medhi

**Affiliations:** ^1^ Postgraduate Institute of Medical Education and Research (PGIMER), Chandigarh, Chandigarh, India

## Abstract

Dementia is a neurodegenerative disorder characterized by cognitive decline, significantly impacting caregivers, especially in collectivist societies like India, where caregiving is viewed as a familial duty. Caregivers experience emotional, social, and financial burdens, which result in work productivity loss, absenteeism, presenteeism, and a reduction in quality of life. Furthermore, the financial strain on caregivers, due to medical costs and reduced employment opportunities, adds to the overall burden. This proposal seeks to address these challenges through the development and implementation of structured, community‐based programs and policy reforms.

This care practice proposal is grounded in the theory of stress management and caregiving burden, emphasizing the need for non‐pharmacological interventions such as stress‐reduction techniques and practical training for caregivers. Structured training programs equip caregivers with the necessary skills to manage dementia patients at home more effectively. These programs will focus on stress management, coping strategies, and improving patient care through non‐pharmacological means. Additionally, support groups to provide emotional relief and reduce the isolation that caregivers often experience.

The proposal also highlights the need for systemic policy reforms, particularly regarding insurance coverage. Comprehensive insurance policies that cover home‐based care, respite services, and dementia‐related medical expenses are crucial in mitigating the financial burden on caregivers. This aspect of the proposal aims to inform policy development by ensuring that families have the financial support they need to manage the long‐term care of dementia patients.

The outcomes of this proposal have the potential to inform future research on caregiver stress and dementia care. By reducing caregiver burden and improving patient care through non‐pharmacological interventions, we hypothesize that both caregiver and patient well‐being can be enhanced. Furthermore, the proposed insurance reforms could reduce out‐of‐pocket expenses, thereby improving the quality of life for caregivers and alleviating economic strain on the healthcare system.

